# EXPLORING CROSS-SECTOR PARTNERSHIPS TO UNDERSTAND DISASTER PREPAREDNESS FOR OLDER AND VULNERABLE POPULATIONS

**DOI:** 10.1093/geroni/igae098.1605

**Published:** 2024-12-31

**Authors:** Leah Haverhals, Lindsay Peterson

**Affiliations:** Chair; Discussant

## Abstract

As disasters continue to increase in frequency and disproportionately impact older adults and other vulnerable populations, more directed focus is needed to facilitate cross-disciplinary partnerships to better support and encourage disaster preparedness and response for these groups. Gerontologists and social scientists have opportunities to partner with engineers, geologists, demographers, epidemiologists, and other community partners to best address gaps in disaster preparation and response in research and practice. This symposium highlights current cross-sector partnerships supporting older adults before, during, and after disasters in various settings across the United States (US). Dr. Tamar Wyte-Lake will explore how interorganizational relationships between the US Department of Veterans Affairs and home health agencies can be leveraged to maintain care across providers during disasters. Emily Killian will describe the Disaster PrepWise (DPW) program, developed through community-engaged processes involving disaster management and public health agencies as well as aging service providers. Dr. Omolola Adepoju will share academic-community partnerships created to build a geospatial-photoethnography dashboard to chronicle and assess successive disaster events on affordable housing and mental health outcomes in Houston. Dr. Holly Dabelko-Schoeny will address how older adults in affordable housing experience extreme weather, and roles and experiences of service coordinators in preparedness and response. Elana Kieffer will describe how older New Yorkers and professionals working with them have demonstrated resilience since the COVID-19 pandemic. Dr. Lindsay Peterson will act as discussant. This is a Disasters and Older Adults Interest Group Sponsored Symposium. Disasters and Older Adults Interest Group Sponsored Symposium

